# Bile metabolites as diagnostic biomarkers for perihilar cholangiocarcinoma

**DOI:** 10.1038/s41598-023-27603-6

**Published:** 2023-02-23

**Authors:** DengYong Zhang, GuanRu Zhao, Wanliang Sun, Dongdong Wang, Shuo Zhou, Zhong Liu, Zheng Lu

**Affiliations:** Department of General Surgery, The First Affiliated Hospital of Bengbu Medical College, No. 287, Changhuai Road, Longzihu District, Bengbu, 233000 Anhui China

**Keywords:** Cancer, Biomarkers, Gastroenterology

## Abstract

It is difficult to directly obtain pathological diagnosis of perihilar cholangiocarcinoma (pCCA). Analysis of bile in the pCCA microenvironment, based on metabolomics and statistical methods, can help in clinical diagnosis. Clinical information, bile samples, blood liver function, blood CA199, CEA, and other indicators were collected from 33 patients with pCCA and 16 patients with gallstones. Bile samples were analyzed using untargeted metabolomics methods. A combination of multivariate and univariate analyses were used to screen for potential differential metabolites Through Kyoto Encyclopedia of Genes and Genomes (KEGG) enrichment and differential metabolite remodeling, we explored changes in the pCCA pathway and potential therapeutic targets. There were significant differences in patient blood TBIL, ALT, AST, TBA, CA19-9, and CEA indices (p < 0.05, |log2(fc)| ≥ 1) between two groups. A significant correlation was found between these different indicators by Spearman's analysis. The clinical parameters were correlated with mass-to-charge ratios of 305 (Positive Ion Mode, POS) and 246 (Negative Ion Mode, NEG) in the metabolic group (|r| ≥ 0.7, P ≤ 10^−7^). The result of this study indicated that bile untargeted metabolomics combined with statistical analysis techniques may be used for diagnose and treatment of pCCA.

## Introduction

The incidence of pCCA accounts for 50–60% of cholangiocarcinomas and is the most common type of cholangiocarcinoma^1^. Surgery is the only cure, and the 5-year survival rate after surgery is less than 19%^2^. The 5-year survival rate for non-operative patients is extremely low. Nowadays, an initial diagnosis of cholangiocarcinoma mainly depends on imaging tests such as CT, MRI, and blood CA199. Acquisition of pathological diagnosis is generally assisted by the following methods: (1) Ultrasound-guided fine-needle aspiration biopsy, which is difficult to carry out due to technical and tumor size constraints. The success rate of tissue acquisition is very low^3,4^. (2) Endoscopic retrograde cholangiopancreatography (ERCP) or EUS tissue biopsy. With pCCA, ERCP has difficulty accessing the upper bile duct, so it is not easy to implement this method. Moreover, for operable patients, it has a risk of implantation metastasis^5–9^. (3) Some scholars have conducted percutaneous transhepatic biliary drainage (PTBD) and then used a choledochoscope to conduct pathological biopsy through the sinus tract. This method demands a high level of technology and equipment, and has a low success rate^10,11^.

The serious drawbacks of all these approaches make it clear that new clinical diagnosis methods for pCCA need to be explored to assist clinical diagnosis. Some researchers have used the differential metabolites between blood of cholangiocarcinoma patients and normal people's blood as the diagnostic basis for cholangiocarcinoma. This method is mainly helpful for the screening of normal patients^12,13^. However, for patients who are clinically suspected to have pCCA based on imaging data, new methods need to be explored to improve the accuracy of diagnosis. Most patients with pCCA suffer from intrahepatic cholestasis due to biliary obstruction. The accumulated bile is a tumor microenvironment substance, which may contain metabolites for better diagnosis of pCCA. Generally, the most prevalent components of bile were metabolites, and the least were DNA, protein, and RNA. We performed metabolomic analysis of bile in an attempt to explore potential metabolites that would aid in clinical diagnosis of pCCA. Bile from patients with pCCA is easily accessible to our department because we performed PTBD therapy on most patients with pCCA, and bile is obtained via PTBD.

## Materials and methods

### Patient cohort and bile collection

We collected the clinical data of 33 patients with pCCA (pCCA group) who were admitted to the First Affiliated Hospital of Bengbu Medical College from January 2021 to January 2022, and 16 patients with gallstones (control group) who were admitted to the hospital during the same period. All 33 patients of pCCA group underwent PTBD. We excluded a patient with pCCA who did not undergo PTBD. Immediately after PTBD, bile was collected and placed into 10 mL test tubes and then stored in a refrigerator at − 80 °C. Bile samples from 16 patients in the control group were collected (after surgical resection or at the time of percutaneous transhepatic gallbladder puncture and drainage (PTGD)) in a 10 mL test tube and stored in a refrigerator at − 80 °C. All samples were obtained with the informed consent of the patient, and this study was approved by the Ethics Committee of Bengbu Medical College (No. 2021230).

Basic information such as height, weight, and age at the time of admission were recorded for all enrolled patients. Meanwhile, blood was drawn to examine liver function indicators (ALB, ALT, AST, CHOL, TBA, TBIL, and TG) and blood tumor indicators (CA19-9 and CEA).

### LC–MS/MS untargeted metabolomics analysis

#### Extraction of metabolites from bile samples

After the samples were slowly thawed at 4 °C, 100 μL was placed in a 1.5 mL Eppendorf tube. After 300 μL of extractant (methanol: ACN = 2:1, v:v, − 20 °C pre-cooled) and 10 μL of internal standard were added, the samples were vortexed and mixed for 1 min, followed by ultrasound in an ice-water bath for 30 min, and then, the samples were allowed to stand for 30 min at − 20 °C and centrifuged at 20,000 rcf for 15 min at 4 °C. After centrifugation, 300 μL of the supernatant was pumped dry in a refrigerated vacuum concentrator, and then 100 μL of reconstituted solution (acetonitrile:H_2_O = 7:3, v:v) was added for reconstitution. The supernatant was vortexed for 1 min, and centrifuged at 4 °C and 20,000 rcf for 15 min. The supernatant was placed in a loading bottle. 10 μL of each sample supernatant was mixed into quality-control (QC) samples to evaluate the repeatability and stability of the LC–MS analysis process.

#### Chromatographic condition

The column was a BEH C18 (1.7 μm, 2.1 × 100 mm, Waters, USA). The POS mobile phases are aqueous 0.1% formic acid (Solution A) and 100% methanol/0.1% formic acid (Solution B). The NEG mobile phases are an aqueous solution containing 10 mM ammonium formate (Solution A) and 95% methanol containing 10 mM ammonium formate (Solution B). The following gradients were adopted for elution: 0–1 min, 2% of solution B; 1–9 min, 2–98% of liquid B; 9–12 min, 98% of liquid B; 12–12.1 min, 98–2% of liquid B; 12.1–15 min, 2% of solution B. The flow rate was 0.35 mL/min, the column temperature was 45 °C, and the injection volume was 5 μL.

#### Mass spectral condition

A Q-Exactive mass spectrometer (Thermo Fisher Scientific, USA) was used for primary and secondary mass spectral data acquisition. The mass-to-nuclear ratio range of the MS scan was 70-1050. The primary resolution was 70,000, the AGC was 3e6, and the maximum injection time was 100 ms. We selected Top 3 for fragmentation based on parent ion intensity, and collected secondary information. The secondary resolution was 17,500, the AGC was 1e5, and the maximum injection time was 50 ms. The fragmentation energy was set to 20 eV, 40 eV, and 60 eV. The parameters for the ion source (ESI) were: sheath-gas flow rate of 40, Aux-gas flow rate of 10, spray voltage (|KV|) of 3.80 in POS mode, and 3.20 in NEG mode. The ion-transport-tube temperature was 320 °C and the aux gas heater temperature was 350 °C. In order to provide more reliable experimental results, we randomly sequenced the samples during instrument detection so as to reduce system error. One QC sample was inserted for every 10 samples.

#### Metabolomics data pre-processing

Raw data collected by LC–MS/MS were imported to Compound Discoverer 3.1 (Thermo Fisher Scientific, USA) for data processing. The primary procedures were peak extraction, intra-and inter-group retention-time correction, addition ion combination, missing value filling, background-peak labeling, and metabolite identification. Finally, we derived the molecular weight, retention time, peak area, and identification result of the compound. The metabolites were identified using a combination of multiple databases including The Human Metabolome Database^14^, MZCloud (https://www.mzcloud.org/), and CHEM Spider (http://www.chemspider.com/). The main parameters for metabolite identification were as follows: precursor mass tolerance of < 5 ppm, Fragment Mass Tolerance < 10 ppm, and retention time tolerance of < 0.2 min. The results obtained by Compound Discoverer 3.1 were imported to metaX for data pre-processing, after which the following procedures were carried out: (1) Probabilistic Quotient Normalization (PQN)^15^ to normalize the data to obtain the relative peak areas; (2) Calibration of batch effects using QC-RLSC (Quality Control-Based Robustness Signal Correction)^16^; (3) Deletion of compounds with a coefficient of variation greater than 30% of the relative peak areas from all QC samples.

### Statistical analysis

We constructed the ROC discriminant model using the Biomarker Analysis module in MetaboAnalyst5.0 (https://www.metaboanalyst.ca/metaboanalyst/upload/ROCUPLOADVIEW.xhtml). After log10 transformation, and Pareto scaling, the data matrix was used for the next analysis. The data was untargeted metabolomics analysis data. The data were processed by log10 transformation and Pareto scaling normalization in Simca 14.1 software. Orthogon Partial Lead Squares Method-Discriminant Analysis (OPLS-DA) model was then constructed using sevenfold cross validation. We verified the validity of the model by 999 permutation tests and conducted Spearman correlation analysis and volcanic map building in Origin lab 2022. Pathway enrichment analysis was performed at the KEGG official website (https://www.genome.jp/kegg/) and reconstructed in Adobe Illustrator 2022. We then transformed the compound matrix by log10 and analyzed it in Graph Pad Prism 8.4.0.

### Institutional review board statement

All procedures followed were in accordance with the Helsinki Declaration of 1975, as revised in 2008 (5) concerning Human. The study was approved by the Ethics Committee of Bengbu Medical College (No. 20211123).

### Informed consent

The informed consent of these patients was obtained.

## Results

### Metabolomics analysis and metabolite classification

**Figure 1:**
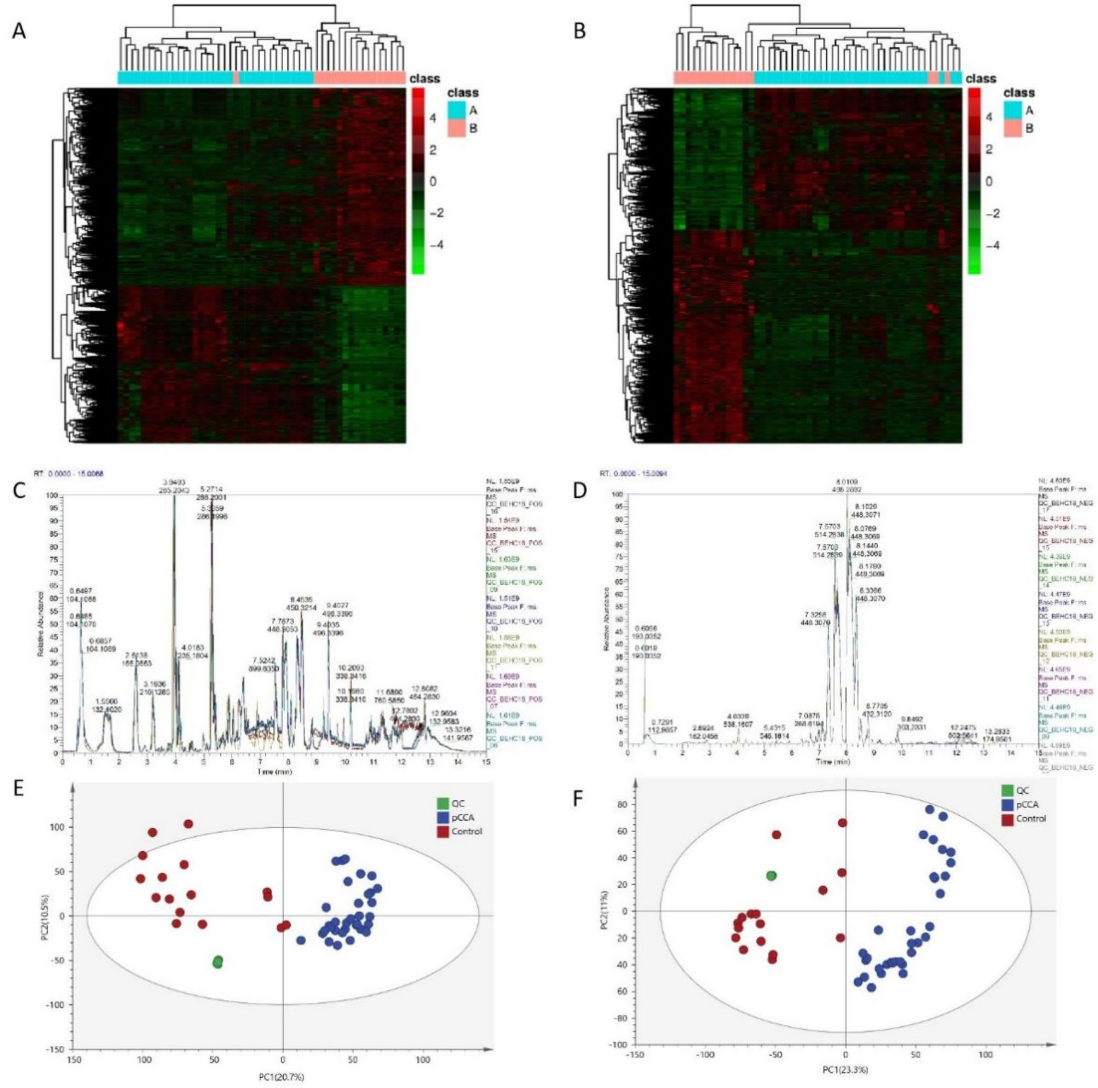
Cluster analysis of differential metabolites. The heatmap of POS (**A**) and NEG (**B**) models. Each row represents a differential metabolite, each column represents a sample, color represents expression levels, green to red corresponding expression levels from low to high (A = pCCA group, n = 33; B = control group, n = 16). The BPC of POS (**C**) and NEG (**D**) models for QC samples. The BPC obtained by continuously delineating the intensity of the strongest ion in the mass spectrum at each time point. The BPCs of all QC samples were overlaid. The spectra overlap well. The fluctuation of retention time and peak response strength was small, indicating that the instrument was in good condition and the signal was stable during the whole sample detection and analysis process. The PCA score map for all samples, in POS (**E**) and NEG (**F**) models. The abscissa is the first principal component PC1, the ordinate is the second principal component PC2, and the ellipse is the 95% confidence interval. Each point represents a sample and the color represents a different group. Numbers in parentheses are the scores for the principal component and represent the percentage of the population variance interpreted by the corresponding principal component (PCCA group, n = 33; Control group, n = 16).

**Figure 2:**
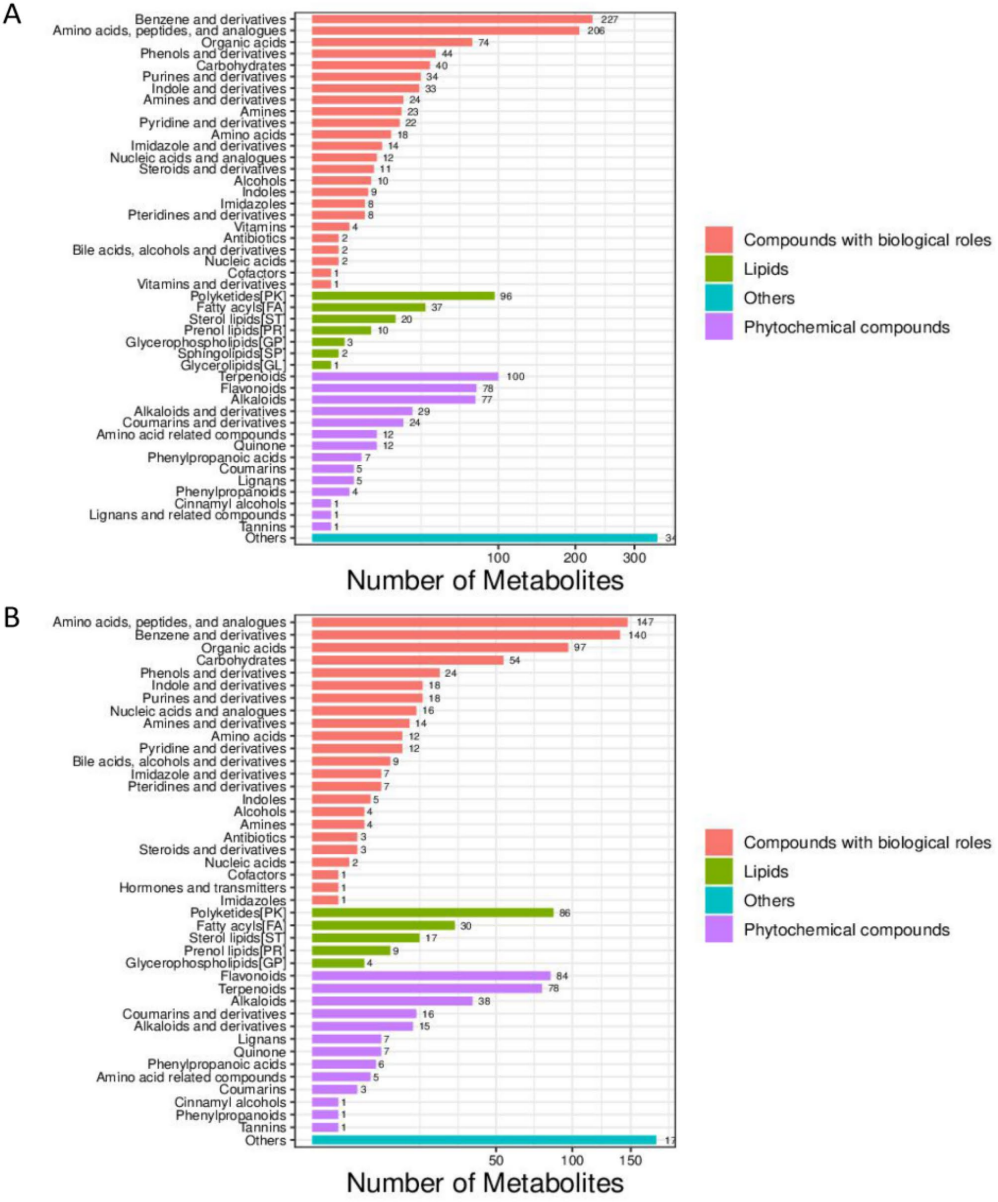
Metabolite classification in POS (**A**) and NEG (**B**) models. The identified metabolites were annotated with classification by reference to the KEGG and HMDB databases. The X axis represented the number of metabolite classifications, and the Y axis represented the metabolite classification items. Others meant that the classification information was the rest of the categories, and the identification results without the classification information were not included in the statistics.

We obtained 14,743 (POS) and 11,688 (NEG) mass-to-charge ratios. These were then identified in the metabolism-related database, yielding 3638 (POS) and 2500 (NEG) substances. We find some difference substances (2746 were down-regulated and 1857 were up-regulated in POS; 2104 were down-regulated and 1679 were up-regulated in NEG) by the criteria: VIP ≥ 1 of the first two principal components of PLS-DA model; Fold-Change ≥ 1.2 or ≤ 0.83; Q-value < 0.05. The heatmap of two groups in POS and NEG model (Fig. 1A,B). BPCs (base peak chromatograms) of all QC samples were overlaid with a suitable spectral overlay. Both the retention time and peak response strength showed low fluctuation, indicating that the instrument was in good condition and the signal was stable throughout the sample-detection and analysis process. The extracted ion chromatogram is shown in Fig. 1C,D. Next, we conducted principal component analysis on samples to reduce the dimensions of the multivariate raw data. In the principal component analysis (PCA) score map, QC samples were grouped together, indicating that the data were reliable (Fig. 1E,F). We conducted classification annotation for the identified metabolites. The classification results showed that the number of metabolites with biological functions was the largest, followed by phytochemical components, lipids, and other components (Fig. 2A,B). The identification results without classification information are not included in the statistics.

### Analysis of clinical indices

**Figure 3:**
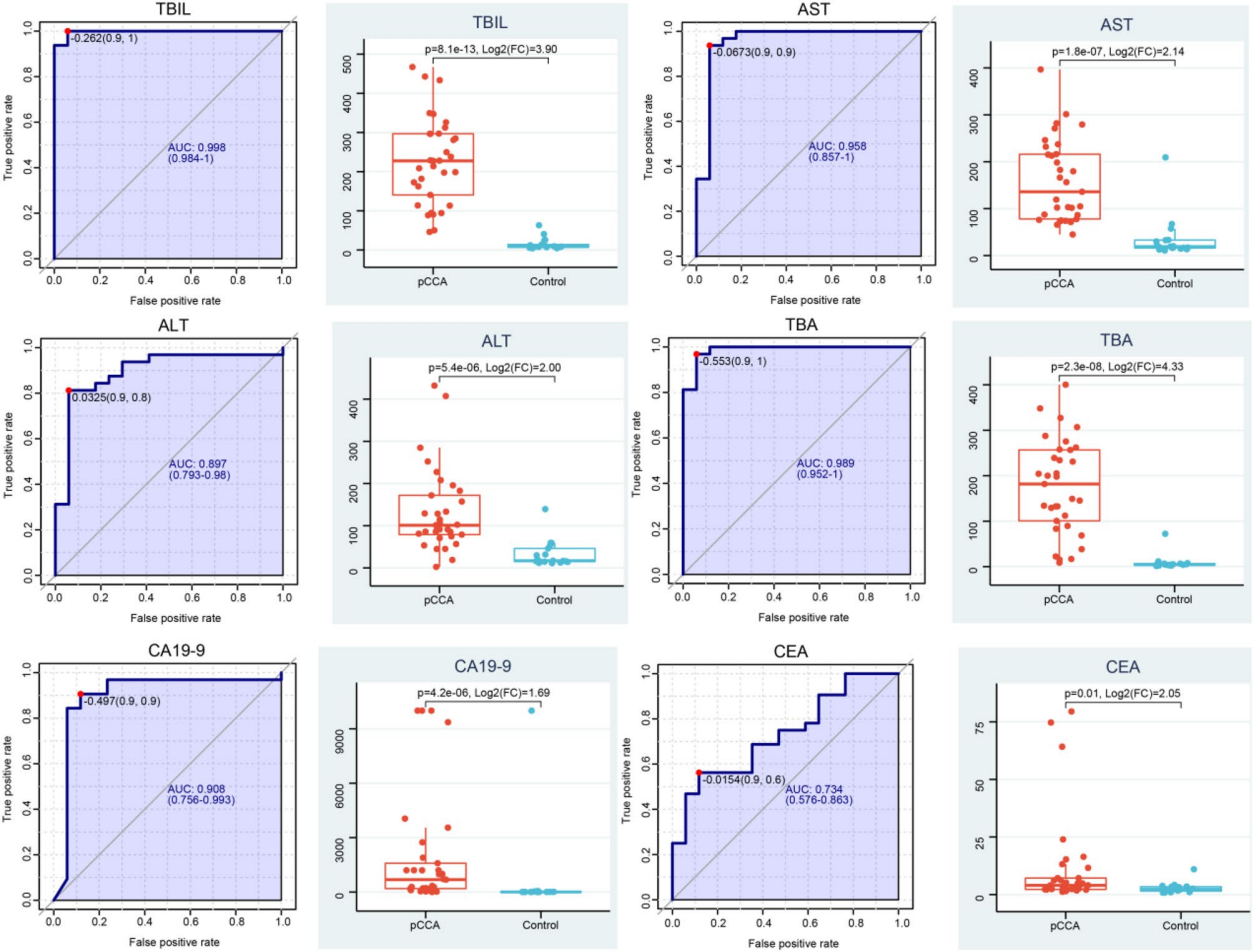
Comparison of clinical indices. There are significant differences in blood TBIL, AST, ALT, TBA, CA19-9,CEA. (p < 0.05, |Log2(pCCA/Control)| ≥ 1). Each point represented one sample. The p < 0.05, |Log2(pCCA/Control)| ≥ 1 indicate significant difference between groups.

**Figure 4:**
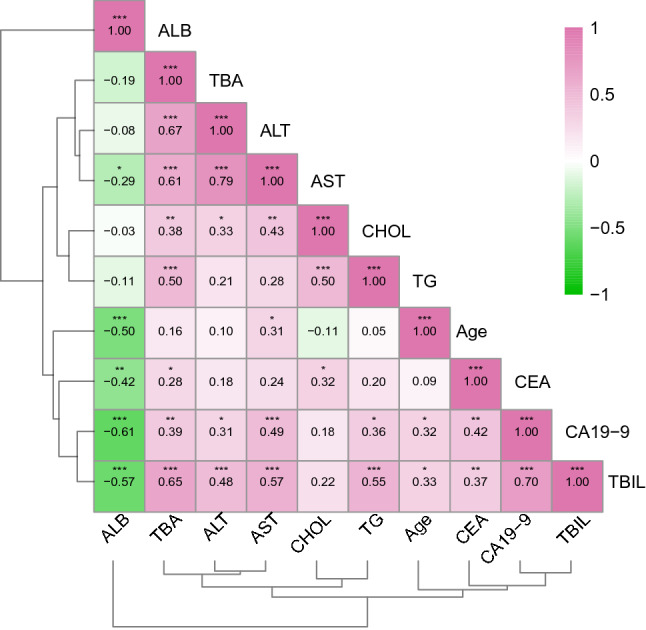
Correlation analysis (Spearman) between clinical indicators. The value [− 1, 1] indicates the degree of correlation, and the closer it is to 1 or − 1 indicates the stronger the correlation. (*p < 0.05, **p < 0.01, ***p < 0.001).

There were significant differences in the following blood indicators between the two groups: TBIL, ALT, AST, TBA, CA19-9, and CEA; and p < 0.05, |Log2(pCCA/Control)| ≥ 1 was selected as the difference standard (Fig. 3). We compared age and blood indicators ALB, CHOL, and TG between the two groups. Although p < 0.05, |Log2(pCCA/Control)| < 1, indicating that there was little difference between these indicators (Fig. S1). Then we analyzed the Spearman correlation among these indicators, and found that there was a strong correlation between ALT and AST, and between TBIL and CA19-9 (r ≥ 0.7, p < 0.05) (Fig. 4). We also analyzed the Spearman correlation between clinical blood indices and the metabolomic matrix, and found that there were strong correlations between clinical blood indices and 305 (POS), 246 (NEG) mass-to-charge ratios (|r| ≥ 0.7, p ≤ 10^−7^). The number of metabolites significantly related to TBIL was the largest (Table 1). Generally speaking, bile and blood TBIL in patients with pCCA were higher. Therefore, it is not difficult to see that metabolites found in the analysis were significantly related to TBIL. This also indirectly suggests that this correlation would be helpful for clinical diagnosis of pCCA.

**Table 1:** Correlation analysis between clinical blood indicators and the number of metabolites (Spearman Analysis, |r| ≥ 0.7). In POS and NEG models, clinical blood indicators are correlated with multiple metabolites, suggesting the potential importance of indicators in the disease process.

	NGE model			POS model		
	Total	Negative correlation	Positive correlation	Total	Negative correlation	Positive correlation
ALT	1	1	0	12	1	11
AST	2	1	1	1	1	0
CA19-9	85	48	37	51	44	7
TBA	31	21	10	90	71	19
TBIL	127	63	64	150	107	43

### KEGG classification and differential metabolite enrichment analysis

**Figure 5:**
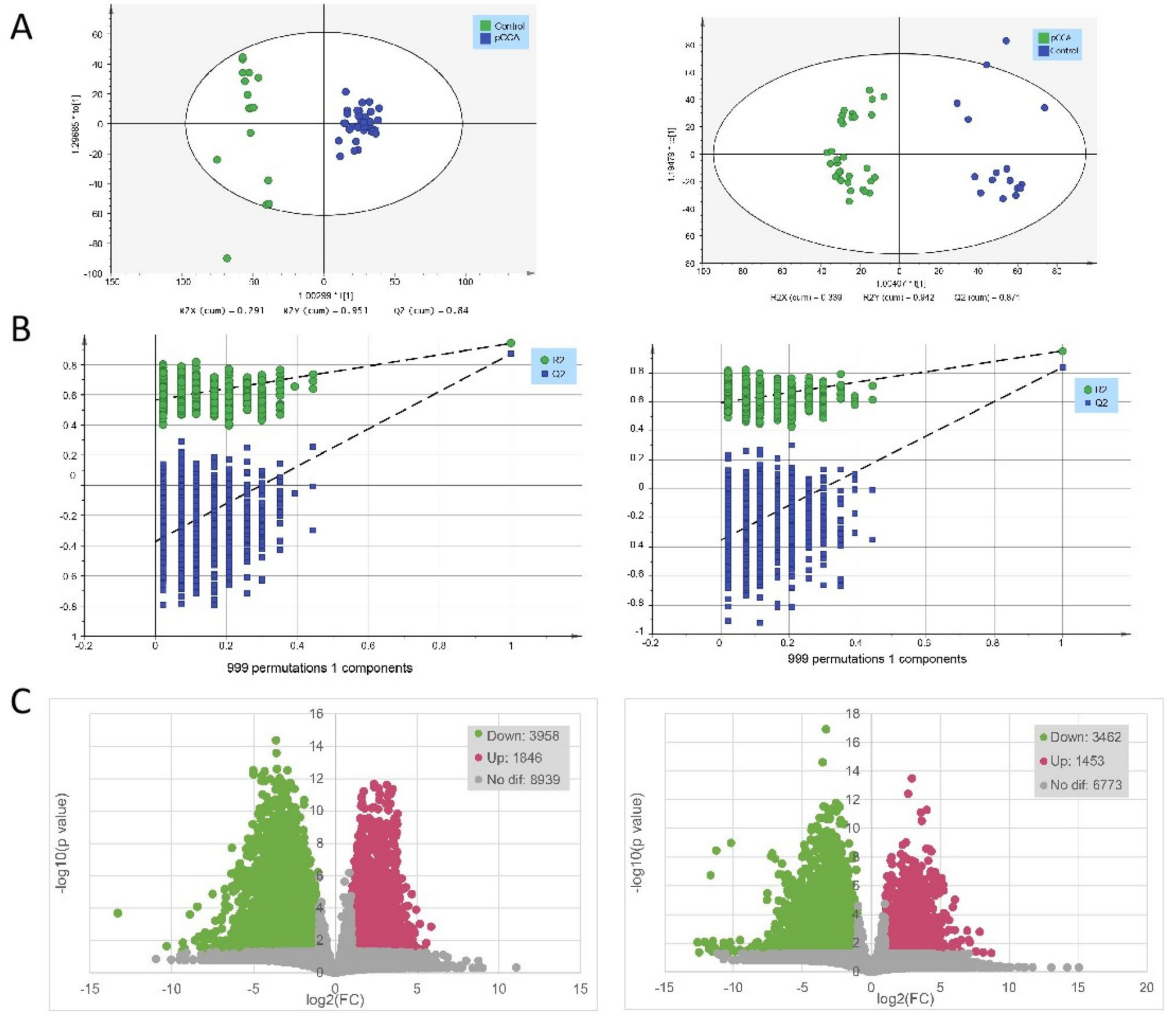
In both POS and NEG models: (**A**) OPLS-DA score plots. The horizontal axis is the first principal component and the vertical axis is the second principal component. The number in parentheses are that scores for that principal component and represent the percentage of population variance explain by the corresponding principal component. (**B**) 999 permutation test result. The two rightmost points were the true R2Y and Q2 values of the model, and the remaining points were the R2Y and Q2 values obtained after the samples were randomly arranged. All predicted values are lower than true values, indicating that the model is true and effective. (**C**) Volcanic map of differential metabolites [p ≤ 0.05, |log2(pCCA/Control)| ≥ 1 considered to be differential]. Blue was the down-regulated differential metabolite, red was the up-regulated differential metabolite, and metabolites with no difference were marked as gray.

**Figure 6:**
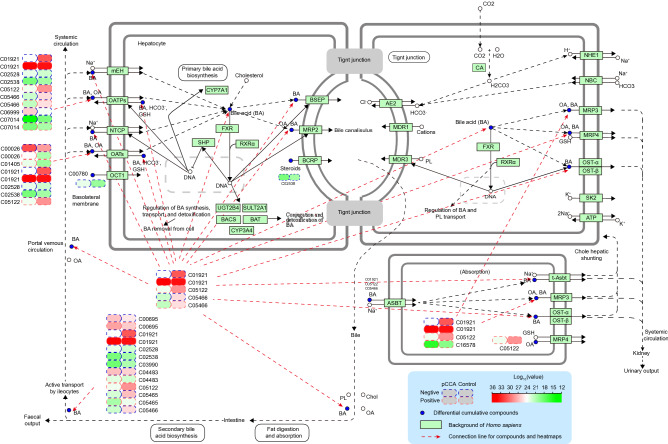
KEGG pathway enrichment analysis of metabolites. Select one of the important pathways shows (bile secretion pathway). The label denotes a potential important target of cholangiocarcinoma discovered in combination with literature review. C01921: Glycocholate; C02528: Chenodeoxycholic acid; C02538: Estrone sulfate; C05466: Glycochenodeoxycholate; C06999: Fexofenadine; C07014: Fluvastatin; C00026: 2-oxoglutaric acid; C01405: Aspirin; C05122: Taurocholic acid; C00695: Cholic acid; C03990: Lithocholic acid; C04483: Deoxycholic acid; C05465: Taurochenodeoxycholic acid; C16578: Morphine 6-beta-d-glucopyranosiduronide.

We performed differential substance screening on the metabolomics data of the two groups. In the OPLS-DA score map, R2Y was 0.951 (POS) and 0.942 (NEG), and Q2 was 0.84 (POS) and 0.871 (NEG). The control group and pCCA group were separated at both ends of the abscissa, indicating that the model differentiation was good (Fig. 5A). We conducted a 999-step permutation test to demonstrate the effectiveness of the model (Fig. 5B). Substances with VIP (variable import in the projection) ≥ 1 based on the OPLS-DA model were used for the next analysis. Univariate analysis was also performed to reduce the false-positive rate of differential substances (Fig. 5C). Based on the volcanic differential mass data (p ≤ 0.05, |log2(pCCA/Control)| ≥ 1) and VIP data, we ultimately considered their intersection to be a differential metabolic component between the pCCA group and the control group. These components were imported to the KEGG website for pathway enrichment analysis. The results showed that multiple metabolites were enriched in the pathways of cofactor biosynthesis, amino acid metabolism, bile secretion, and steroid hormone biosynthesis. The main components of bile, such as bile acids, play an important role in the occurrence, development, diagnosis, and prognosis of cholangiocarcinoma^17,18^. Visual analysis of this pathway showed that the accumulation of multiple substances in the control group was significantly higher than that in the cancer group, e.g., C01921, C05122, and C05466. A few substances had higher accumulation in the pCCA group, e.g., C02528, C06999, and C00780 (Fig. 6). The discovery of these key differential metabolites will provide a reference for future research on the therapeutic targets of pCCA.

## Discussion

The 5-year survival rate of pCCA is low due to its particular anatomical location, low surgical resection rate, and the lack of good chemotherapy or targeted therapeutic drugs. The best way to obtain an accurate diagnosis in a minimally invasive manner for non-surgical patients is still a difficult problem^2,19^. At present, the means for invasive operation to obtain pathology include tissue biopsy guided by ultrasound (US), contrast EUS (enhanced ultrasound), EUS, ERCP, and CT. However, due to the high equipment requirements, advanced skill requirements for operating physicians, and low acquisition positive rate, it has not become widespread in clinical use^20–22^.

Some scholars have proposed a strategy of liquid biopsy for cholangiocarcinoma. Blood metabolomics analyses have revealed differential metabolites that could help to differentiate the diagnosis of intrahepatic cholangiocarcinoma, primary liver cancer, and sclerosing cholangitis. This provides a new idea for minimally invasive diagnosis of cholangiocarcinoma. Some researchers believe that the combination of a bile proteomics test and blood CA199 level in patients with cholangiocarcinoma can help to improve diagnostic accuracy^12^. One group performed transcriptome sequencing analysis on the blood and urine of patients with cholangiocarcinoma^23^. Identified RNA products can also help improve the accuracy of cholangiocarcinoma diagnosis^24^. However, the results of these studies cannot meet clinical needs. So far, no diagnostic markers with high specificity have been found for pCCA and none of the bile samples used came from the tumor microenvironment. It is generally known that one important means of non-surgical treatment for patients with pCCA is biliary stent implantation. In our center, biliary stents are placed through PTBD. At the same time, for some pCCA patients with jaundice who have undergone surgical resection, PTBD is routinely used to reduce jaundice before surgery^25^. It is easy to obtain bile in the tumor microenvironment during PTBD. Compared with hematological examination, tumor markers in bile are more interesting, because they are microenvironment substances that are in direct contact with tumors.

According to our metabolomics analysis, the metabolites related to biological functions are the most abundant. By analyzing the clinical information for the two groups, we found that there were significant differences in blood TBIL, ALT, AST, TBA, CA19-9, and CEA between them. The difference in blood TBIL is due to the increase in TBIL caused by biliary obstruction in patients with pCCA, and the abnormal changes in ALT, AST, and TBA are caused by secondary abnormal liver function. The differences in CA19-9 and CEA are more helpful for diagnosis of pCCA. Spearman correlation analysis among the clinical indices also revealed that ALT had a strong correlation with AST, while TBIL was strongly correlated with CA19-9. In general, when liver function is abnormal, changes in ALT and AST are related. However, changes in TBIL and CA19-9 are generally not related. In our research, we found that they have a strong correlation, which may be an important basis for the diagnosis of pCCA. We speculate that patients with a strong correlation between TBIL and CA19-9 are more likely to be diagnosed with pCCA. We hope that these models will be useful for clinical diagnosis of pCCA in the future. The approach is similar to that used for clinical diagnosis of hepatocellular carcinoma, with which the next drug treatment can be carried out without obtaining a pathological diagnosis.

In general, the occurrence and development of cholangiocarcinoma are closely related to abnormal metabolic changes. We imported components of bile metabolomics into the KEGG website for pathway enrichment analysis. The results showed that multiple metabolites were enriched in the bile secretion and bile acid metabolism pathways. Then we visually analyzed this pathway and found low accumulation of multiple substances in the pCCA group, such as C01921, C05122, and C05466, and high accumulation of a few substances in the pCCA group, such as C02528, C06999, and C00780. Abnormalities of metabolites arise from dysfunction of proteins. Previous reports have pointed out that multiple key molecules (on our signaling pathway, Fig. 6) play a regulatory role in the development of cholangiocarcinoma.

CYP3A4, a P450 enzyme, has been confirmed to play a role in the occurrence and development of various tumors, for example in oxygen metabolism of liver cancer and progression of breast and prostate cancer^26,27^. An intrahepatic cholangiocarcinoma model with hepatocyte differentiation showed higher CYP3A4 activity and bile acid production^28^. It may also play an important role in the metabolism of cholangiocarcinoma, but this requires further laboratory verification^29^. Infigratinib (INF), a fibroblast growth factor receptor inhibitor, is being used in clinical studies for advanced cholangiocarcinoma. INF mediates inactivation of CYP3A4 by irreversible covalent addition of hemin and/or apoprotein, thereby exerting an inhibitory effect on cholangiocarcinoma^30^. FXR (farnesoid x receptor, also known as Nuclear Receptor Sub Family 1 Group H Member 4,NR1H4) is a nuclear receptor that can be activated by bile acid, and after activation, it can promote excretion of bile acid and inhibit uptake of bile acid in hepatocytes. FXR is also involved in the regulation of genes associated with lipid and carbohydrate metabolism. Activation of FXR promotes anti-inflammatory and anti-fibrotic effects. Some FXR agonist drugs have shown good efficacy in the treatment of pathological cholestatic diseases including primary cholestatic diseases, cholangitis, primary sclerosing cholangitis, and cholangiocarcinoma^31,32^. NTCP is also called SLC10A1 (solute carrier family 10 member 1). Expression of SLC10A1 in rat cholangiocarcinoma tissues is reduced, and this is related to the proliferation phenotype of cholangiocarcinoma, which may be one of the therapeutic targets of cholangiocarcinoma^33^. Bsep (bile salt export pump), also known as ABCB 11 (ATP binding cassette sub family B member 11), is a key factor in maintaining the homeostasis of bile salt metabolism. A lack of ABCB11 increases bile acid production and promotes the formation of intrahepatic cholangiocarcinoma, and it also affects the action of FXR molecules^34^. Bsep has also been shown to be a downstream gene of FXR^35^. Expression of ABCB11 in rat pCCA is significantly reduced, and its expression is closely linked to the metabolic process of cholesterol and bilirubin. These results suggest that targeted intervention with Bsep may be a new strategy to explore for the treatment of pCCA^36^.

## Conclusions

Based on our literature analysis, we believe that metabolomics analysis of bile will facilitate the establishment of clinical diagnostic models for cholangiocarcinoma, as well as exploration of potential therapeutic targets of cholangiocarcinoma. These results will provide a theoretical basis for the subsequent experimental verification of cholangiocarcinoma cytology.

## Supplementary Information

Supplementary Figure S1.

## Data Availability

The data generated and/or analyzed during this study are available from the corresponding author on reasonable request.
