# Supplementary material for: Bile metabolites as diagnostic biomarkers for perihilar cholangiocarcinoma
Source: Sci Rep. 2023 Feb 23;13:3177. doi: 10.1038/s41598-023-27603-6 (PMC9950048; doi:10.1038/s41598-023-27603-6)
Supplement: Supplementary file 1 — Supplementary Figure S1. [file 41598_2023_27603_MOESM1_ESM.docx]

**Bile metabolites as diagnostic biomarkers for perihilar cholangiocarcinoma**

DengYong Zhang^†1^, GuanRu Zhao^†1^, Wanliang Sun^1^, Dongdong Wang^1^, Shuo Zhou^1^, Zhong Liu, Zheng Lu^1^*

**Supplementary Materials:**

**Figure S1.** Comparison of differences in clinical indicators of age, ALB, CHOL, and TG between the two groups（|Log2(pCCA/Control)|<1）.

**
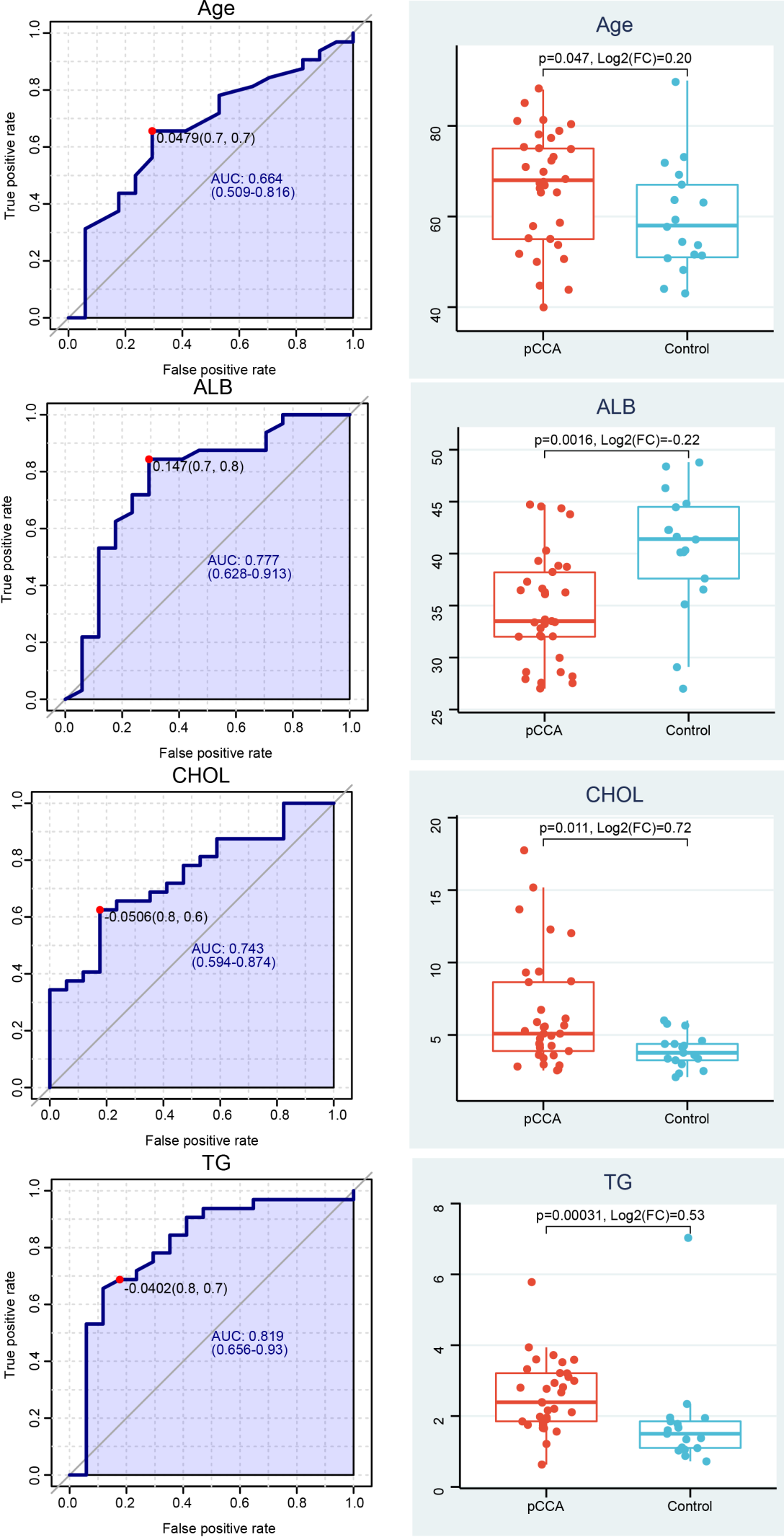
**
